# Including irrigation in niche modelling of the invasive wasp *Vespula germanica* (Fabricius) improves model fit to predict potential for further spread

**DOI:** 10.1371/journal.pone.0181397

**Published:** 2017-07-17

**Authors:** Marelize de Villiers, Darren J. Kriticos, Ruan Veldtman

**Affiliations:** 1 Department of Conservation Ecology and Entomology, Stellenbosch University, Stellenbosch, South Africa; 2 CSIRO, Canberra, Australia; 3 The University of Queensland, St. Lucia, Queensland, Australia; 4 South African National Biodiversity Institute, Cape Town, South Africa; University of Thessaly School of Agricultural Sciences, GREECE

## Abstract

The European wasp, *Vespula germanica* (Fabricius) (Hymenoptera: Vespidae), is of Palaearctic origin, being native to Europe, northern Africa and Asia, and introduced into North America, Chile, Argentina, Iceland, Ascension Island, South Africa, Australia and New Zealand. Due to its polyphagous nature and scavenging behaviour, *V*. *germanica* threatens agriculture and silviculture, and negatively affects biodiversity, while its aggressive nature and venomous sting pose a health risk to humans. In areas with warmer winters and longer summers, queens and workers can survive the winter months, leading to the build-up of large nests during the following season; thereby increasing the risk posed by this species. To prevent or prepare for such unwanted impacts it is important to know where the wasp may be able to establish, either through natural spread or through introduction as a result of human transport. Distribution data from Argentina and Australia, and seasonal phenology data from Argentina were used to determine the potential distribution of *V*. *germanica* using CLIMEX modelling. In contrast to previous models, the influence of irrigation on its distribution was also investigated. Under a natural rainfall scenario, the model showed similarities to previous models. When irrigation is applied, dry stress is alleviated, leading to larger areas modelled climatically suitable compared with previous models, which provided a better fit with the actual distribution of the species. The main areas at risk of invasion by *V*. *germanica* include western USA, Mexico, small areas in Central America and in the north-western region of South America, eastern Brazil, western Russia, north-western China, Japan, the Mediterranean coastal regions of North Africa, and parts of southern and eastern Africa.

## Introduction

*Vespula germanica* (Fabricius) (Hymenoptera: Vespidae), known as the European wasp in Australasia, South Africa and South America, and the German wasp elsewhere, is of Palaearctic origin, occurring naturally in Europe, northern Africa and Asia, and being introduced into the USA, Canada, Chile, Argentina, Iceland, Ascension Island, South Africa, Australia and New Zealand [1–4]. Its scavenging feeding habits and use of anthropogenic structures for nesting sites brings the species in close contact with humans [5, 6]. This may lead to a close association between its distribution and areas of human settlements, which may also influence its invasive potential.

*Vespula germanica* is polyphagous, with the adults feeding on carbohydrates in the form of sugars, and workers collecting protein for the larvae [2, 7–9]. Therefore, they can cause a problem for various human economic and recreational activities. For example, in New Zealand, they became a serious problem for beekeeping, robbing the hives of honey and bees [10,11]. In Israel, they were found to damage the udders and teats of dairy cows [12,13]. They also cause problems in orchards and vineyards where they feed on the fruit, and due to their aggressive nature, they attack and sting human workers, especially at harvest [11, 14–16]. Being venomous, they deliver a painful sting and can produce multiple stings, which may result in allergic reactions [14, 15]. Due to their scavenging behaviour, association with human food sources (e.g. protein food consumed by humans, bread and sugary liquids), and aggressive nature, they become a problem at picnic sites, schools and other public places, where they are a nuisance and pose a threat to humans and their pets [6, 15–17]. In addition to anthropogenic impacts, *V*. *germanica* can also impact natural ecosystems in invaded areas [18, 19], where they prey on and compete with a wide range of arthropods [15, 18]. In New Zealand for example, they compete with native species such as birds and invertebrates for honeydew excretions in beech forests, before being displaced by the common wasp, *Vespula vulgaris* (L.) [20, 21].

*Vespula germanica* generally has an annual life cycle, with queens hibernating in sheltered areas through cold winters. During warmer spring weather, the fertilized queens build nests and start new colonies [7, 8, 22, 23]. Nests are mostly built underground, but they may also use tree trunks, cavities in walls, or ceilings of buildings [6, 7, 14, 24–27]. Nests grow in size throughout summer, with workers attending to the larvae, reaching maximum size during autumn [7]. During this time, new queens and increasingly more males are produced, which then mate, and subsequently the queens hibernate during winter while the rest of the colony dies out [7, 27, 28]. However, the phenology of the wasp differs in areas of its invasive range such as Australia and New Zealand where the winters are warmer and the summers are longer than in the higher latitudes in the Northern Hemisphere. In such regions, queens and workers are able to survive the winter period to continue into the next season, resulting in the development of much larger nests [26, 27, 29–31].

From the list of countries it has invaded, it is clear that *V*. *germanica* has a high invasive potential, and can tolerate or adapt to a wide range of habitats and climates. For example, in Argentina, it has spread throughout most of the Patagonian and Sub-Antarctic biogeographical provinces, as well as the southern part of the Monte province [32], while in the USA it now occurs in almost 30 states [4]. Within six years after its introduction in New Zealand, it infested 80 000 km^2^ to inhabit both main islands [15]. In contrast to its behaviour in other invaded countries where it has spread and invaded larger areas, this has not been observed in South Africa. Despite being detected as early as 1974 in the Cape Peninsula [7], *V*. *germanica* currently still only occurs in a relatively small portion of the Western Cape Province [33]. It is unclear why it showed limited invasive behaviour in South Africa and its limited spread has resulted in few studies being conducted locally on the species, with no attempts being made to eradicate the species from the Western Cape [2]. This situation has changed recently with active research being undertaken to establish the feasibility of either eradication, or at least monitoring and preventing further spread at the range edge [33, 34]. The potential distribution of this species and the extent of areas of favourable climatic conditions is important information required to assist such management decisions.

CLIMEX models [35, 36] have been constructed previously to estimate the potential distribution of *V*. *germanica*. The first model was that of Spradbery & Maywald [37], investigating the potential distribution in Australia. The second was done by Tribe & Richardson [38], investigating the potential spread in South Africa. The model by Spradbery & Maywald [37] was updated by Bob Sutherst (CSIRO, Canberra, Australia) during 2004. This updated version was not published, but a CLIMEX parameter file was created in CLIMEX version 3, which is accessible to CLIMEX users [39]. Tribe & Richardson [38] estimated the fynbos biome of the southern and western part of the Western Cape Province to be only marginally suitable, which may explain its low rate of spread. However, these models were built on general data on the presence of *V*. *germanica* in Europe, Asia, North Africa and, in the case of Spradbery & Maywald [37], also the Middle East. Since then, *V*. *germanica* has expanded its distribution, with more presence and absence data in the form of specific geographical point locations becoming available. This makes it possible to update the Sutherst model.

In this paper, we used distribution data for *V*. *germanica* from Argentina and Australia, as well as seasonal phenology data from Argentina to fit a CLIMEX Compare Locations model. The CLIMEX model was then applied to global climate data and the results compared with global presence data (including detailed presence data from South Africa) for *V*. *germanica* in regions not used for model fitting. The validated model was then used to create a global climatic risk map as a composite of natural rainfall and irrigation scenarios.

## Materials and methods

### Distribution data

**Fig 1:**
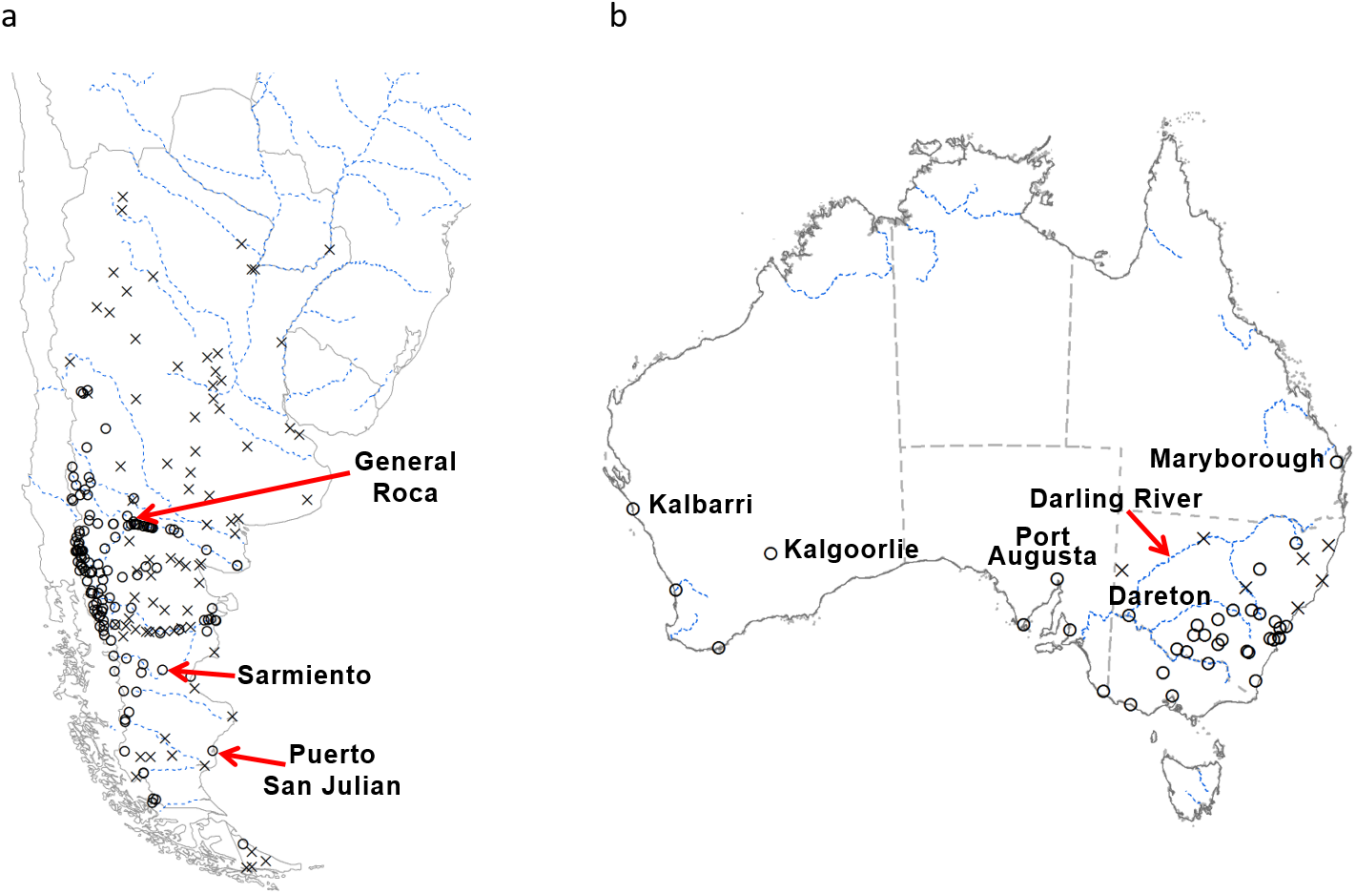
**The distribution of *V*. *germanica* in (a) Argentina and (b) Australia, plus key locations in each country used to model the potential distribution**. For Australia not all presence sites are shown, focusing more on sites in the northern boundary of its distribution. Distribution data for Tasmania is not included as it occurs widespread throughout the island. Open circles: presence sites; black crosses: absence sites; blue dotted lines: main rivers.

Distribution data for Argentina was obtained from Masciocchi & Corley [32] and Maitè Maschiocchi (pers comm.) (Fig 1A), while Australian data was obtained from Spradbery & Maywald [37], Horwood et al. [40], Philip Spradbery (pers comm.) and Marc Widmer (pers comm.) (Fig 1B). South African distribution data was obtained from Haupt [33]. Information on global distribution was obtained from CABI [4].

### CLIMEX

CLIMEX (Hearne Scientific Software Pty Ltd, Australia) [35, 36] is a semi-mechanistic modelling package that was developed mainly to estimate the potential distribution of invasive species, and to explore the climatic factors that influence population growth or decline. The CLIMEX Compare Locations model simulates the mechanisms that influence a species’ population growth and survival responses to climate, in order to estimate its potential geographical distribution and seasonal abundance [36].

CLIMEX assumes that a population may experience two types of season annually, those favourable for growth and those that are stressful, during which the population will decline [35, 36]. The programme integrates a population’s weekly responses to climate and uses these to calculate a number of annual and weekly indices, including annual and weekly Growth Indices (GI_A_ and GI_W_ respectively), stress indices (SI) and the Ecoclimatic Index (EI), which indicates the overall climatic favourability [36]. In addition, stress functions can be fitted for cold, dry, hot, wet, cold-dry, cold-wet, hot-dry and hot-wet stress indices. Besides the temperature and moisture stresses, the potential distribution of a species may also be limited by a minimum length of the growing season measured in degree-days.

The annual Growth Index (GI_A_) represents the potential for population growth and development, and combines the organism’s response to temperature, soil moisture and, where relevant, day-lengths and diapause. CLIMEX combines the growth and stress indices into an overall Ecoclimatic Index (EI), ranging from 0 to 100 [36]. Assigning classes of suitability to EI values between 0 and 100 is usually an arbitrary process intended to reduce the perceived level of model precision compared with that implied by a percentile score.

CLIMEX can provide the user with maps of annual summary variables, such as the Ecoclimatic Index (EI), the annual Growth Index (GI_A_) and the stress indices, as well as weekly time-series graphs of state variables such as the weekly Growth Index (GI_W_) [36]. The CLIMEX model was constructed by iteratively fitting the stress parameters until the geographical distribution simulated by CLIMEX (EI≥1) coincided with the Argentinean and Australian distribution (Fig 1A and 1B), and Argentinean seasonal phenology accorded with graphs of GI_W_ (see Table 1 for parameters). Relevant biological information (e.g. developmental thresholds) informed the selection of the stress mechanisms parameter value selection to ensure that they were biologically plausible.

**Table 1:** CLIMEX parameters used to model the distribution of *V*. *germanica*, based on its distribution in Argentina and Australia, as well as seasonal phenology in Argentina.

Index	Parameter	Value^#^			
		Spradbery	Tribe &	Sutherst	Current
		& Maywald	Richardson	et al.	model
**Temperature**	DV0 = lower threshold (°C)	10	10	10	6.5
	DV1 = lower optimum temperature (°C)	18	18	18	18
	DV2 = upper optimum temperature (°C)	26	26	26	26
	DV3 = upper threshold (°C)	33	31	31	33
**Moisture**	SM0 = lower soil moisture threshold	0	0.2	0.2	0.2
	SM1 = lower optimum soil moisture	0.6	0.8	0.6	0.6
	SM2 = upper optimum soil moisture	1.5	2	1.5	1.5
	SM3 = upper soil moisture threshold	2.5	3	2.5	2.5
**Cold stress**	DTCS = degree-day threshold (stress accumulates if the number	10	10	10	10
	of degree-days above DVCS is below this value) (°C-days)				
	DHCS = stress accumulation rate (week ^-1^)	-0.00014	-0.00014	-0.00014	-0.00016
	DVCS = developmental temperature threshold (°C)	10	10	10	6.5
**Heat stress**	TTHS = heat stress temperature threshold (°C)	31	30	31	33
	THHS = stress accumulation rate (week ^-1^)	0.0035	0.005	0.0035	0.0035
**Dry stress**	SMDS = soil moisture dry stress threshold	0.2	0.2	0.15	0.2
	HDS = stress accumulation rate (week ^-1^)	-0.006	-0.01	-0.008	-0.008
**Wet stress**	SMWS = wet stress threshold	2.5	3	2.5	0
	HWS = stress accumulation rate (week ^-1^)	0.002	0.002	0.002	0
**Hot-wet**	TTHW = hot-wet stress temperature threshold (°C)	26	0	26	22
**stress**	MTHW = hot-wet stress moisture threshold	0.8	0	0.8	0.4
	PHW = stress accumulation rate (week ^-1^)	0.03	0	0.03	0.009
**Annual heat**	PDD = number of degree-days above DV0				
**sum**	needed to complete one generation (°C-days)	350	350	350	350

Parameters for models by Spradbery & Maywald [37], Tribe & Richardson [38] and Sutherst et al. [39] are also included.

^#^Values without units are dimensionless indices of a 100 mm single bucket soil moisture model (0 = oven dry, 1 = field capacity).

The 10’ CliMond climate dataset was used within CLIMEX to represent current climate [41] The CM10_1975H_V1.2 dataset of historical long-term monthly climate averages for minimum and maximum temperature, precipitation and relativity humidity at 09h00 and 15h00 is centred on 1975.

### CLIMEX parameter fitting

The parameter sets of the CLIMEX models by Spradbery & Maywald [37], Tribe & Richardson [38] and Sutherst et al. [39], were taken as a starting point when building the *V*. *germanica* model. These models were constructed based on the known distribution of the wasp in Europe, Asia, the Middle East and North Africa [37, 38]. The model based on the parameter set of Spradbery & Maywald [37] indicated that wasp populations benefit from cool to hot, dry conditions, but are sensitive to prolonged cold, as well as hot, wet conditions. These previous models estimated certain parts in the dry desert such as Patagonia in Argentina, where *V*. *germanica* is known to occur, to be climatically unsuitable [32, 37–39]. While climate is the primary range-limiting factor for poikilotherms [42, 43], climate-modifying factors such as irrigation can also play an important role in extending a species range beyond the limits afforded by climate. We therefore hypothesised that the persistence of *V*. *germanica* in these xeric regions may be predicated on irrigation. Google Earth revealed that many of these presence sites in Argentina lie alongside river beds. We explored the possibility that consideration of irrigation patterns provides a better model fit. Further adjustments were made to the model to accord with published information on the temperature thresholds for *V*. *germanica*, as well as distribution data from Argentina and Australia (Fig 1A and 1B), and phenological observations in Argentina (Table 1).

#### Temperature index

The temperature index parameters were similar to those used in the models by Spradbery & Maywald [37], Tribe & Richardson [38] and Sutherst et al. [39] (Table 1). The minimum temperature for development (DV0) was reduced from 10°C to 6.5°C to allow all presence sites along the Andes mountains in Argentina [32] to be modelled as suitable. This was also in line with results from Coelho & Ross [44] and Kasper et al. [23], indicating that 7°C was the lower threshold for *V*. *germanica* activity, as well as Goller & Esch [45], which stated the lower threshold for flight activity to be 6 to 7°C. The lower (DV1) and upper (DV2) optimum temperature was kept at 18°C and 26°C respectively. The maximum temperature for development (DV3) was set to 33°C, which was in line with the upper threshold of 35°C for activity, determined by Coelho & Ross [44]. Austin & Hopkins [46] and Kasper et al. [23] also recorded a decrease in wasp activity for temperatures above 35°C. The number of degree-days per generation (PDD) was kept at 350. Given the reduced value of DV0, this indicated a slightly higher thermal sum for a generation compared with previous models.

#### Moisture index

The same moisture thresholds of Sutherst et al. [39] were used. The lower moisture threshold (SM0) was set to 0.2, somewhat above permanent wilting point. The lower optimal soil moisture threshold was set to 0.6. The upper optimal soil moisture threshold (SM2) and limiting high soil moisture threshold (SM3) was set to 1.5 and 2.5 respectively. A similar parameter set was also used by Spradbery & Maywald [37].

#### Cold stress

A similar cold stress scenario to that of Spradbery & Maywald [37], Tribe & Richardson [38]) and Sutherst et al. [39] was used. The degree-day threshold (DTCS) value was kept at 10°C-days and the stress accumulation rate was increased from -0.00014 week^-1^ to -0.00016 week^-1^ to compensate for the lower DV0 value. The developmental temperature threshold (DVCS) was also decreased from 10 to 6.5°C to be in line with the lower DV0 value.

#### Heat stress

The heat stress mechanism was the same as that used by Spradbery & Maywald [37] and Sutherst et al. [39]. However, the heat stress temperature threshold (TTHS) was increased from 31 to 33°C to accommodate the higher DV3 value in the current model. The heat accumulation rate (THHS) was kept the same at 0.0035 week^-1^.

#### Dry stress

The soil moisture dry stress threshold (SMDS) was set to 0.2, and the stress accumulation rate (HDS) to -0.008 week^-1^. This resulted in the drier areas of Patagonia, as well as the drier areas in the northern boundary of its distribution in southern Australia, e.g. Kalgoorlie (Western Australia), Port Augusta (South Australia) and Dareton (New South Wales) being modelled as unsuitable, where persistence of *V*. *germanica* is more plausibly contingent on irrigation.

#### Wet stress

Wet stress was not included in the model, as it had minimal impact on the modelled potential distribution.

#### Hot-wet stress

The hot-wet stress parameters of Spradbery & Maywald [37] and Sutherst et al. [39] are considered to be too high since hot-wet stress generally reflects the effects of competition, predation or parasitism, and hence the stress tends to accumulate over longer periods compared with hot or wet stress functions [36]. This means that it should have a low accumulation rate. The hot-wet temperature threshold (TTHW) was set to 22°C, the hot-wet moisture threshold (MTHW) to 0.4 and the stress accumulation rate (PHW) to 0.009 week^-1^. This estimated south-east Asia to be climatically unsuitable, which corresponds to the absence of *V*. *germanica* from these areas [4].

#### Seasonal phenology

Seasonal phenology trends for the Patagonian region in Argentina were obtained from Maitè Masciocchi (pers comm.). In this region, the queens start the colonies during late September to early October. The first workers are seen in January, showing a peak in abundance during March. By late April or early May the wasps disappear completely. This was compared graphically with the GI_W_ values from the model output to see whether or not there was concordance between the modelled GI_W_ and the seasonal occurrence throughout the year. Where there was a mismatch, the inclusion of irrigation was explored to see if a better fit could be obtained.

#### Irrigation

A summer top-up irrigation scenario of 2.5 mm day^-1^ was applied to produce two types of maps: (a) a map showing the estimated distribution with the assumption that all areas across the world are irrigated, and (b) a risk map contingent on irrigation being practiced in the 10’ cell according to the global irrigation map [47], producing a composite risk map. In areas that were under irrigation according to Siebert et al. [47], the EI of the irrigation scenario was mapped, while in areas where zero irrigation is applied, the EI of the non-irrigation scenario was mapped.

## Results

**Fig 2:**
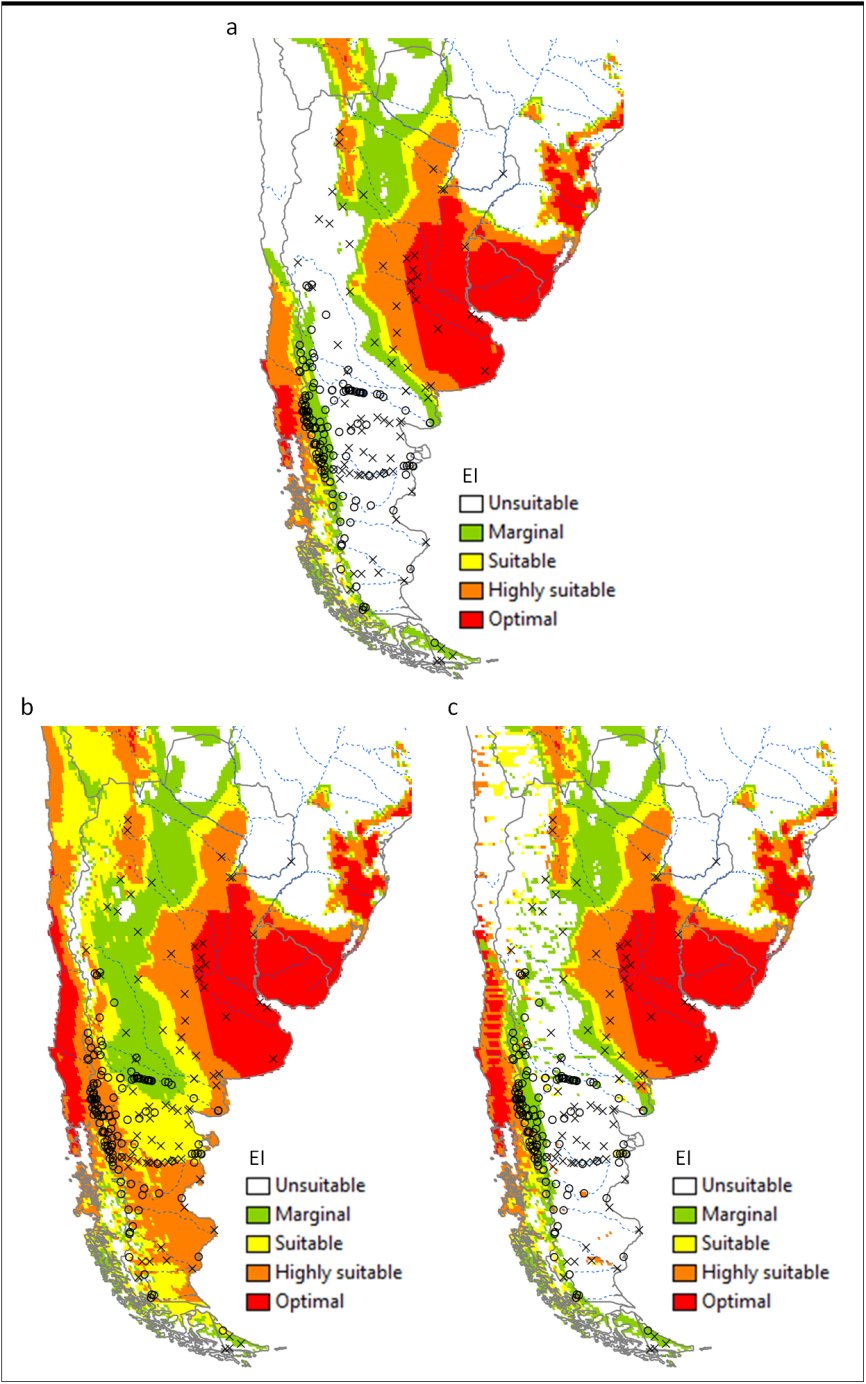
**The projected climate suitability for *V*. *germanica* in Argentina, (a) without irrigation, (b) with 2.5 mm day**^**-1**^ **top-up irrigation during summer and (c) with a composite risk irrigation scenario (where areas are not under irrigation, the EI of the natural rainfall scenario is mapped, while with areas under irrigation the EI of the irrigation scenario is mapped), using the CLIMEX Ecoclimatic Index (EI).** Open circles: presence sites; black crosses: absence sites; blue dotted lines: main rivers. Unsuitable: EI = 0; marginal: EI = 1–4; suitable: EI = 5–9; highly suitable: EI = 10–29; optimal: EI = 30–100.

**Fig 3:**
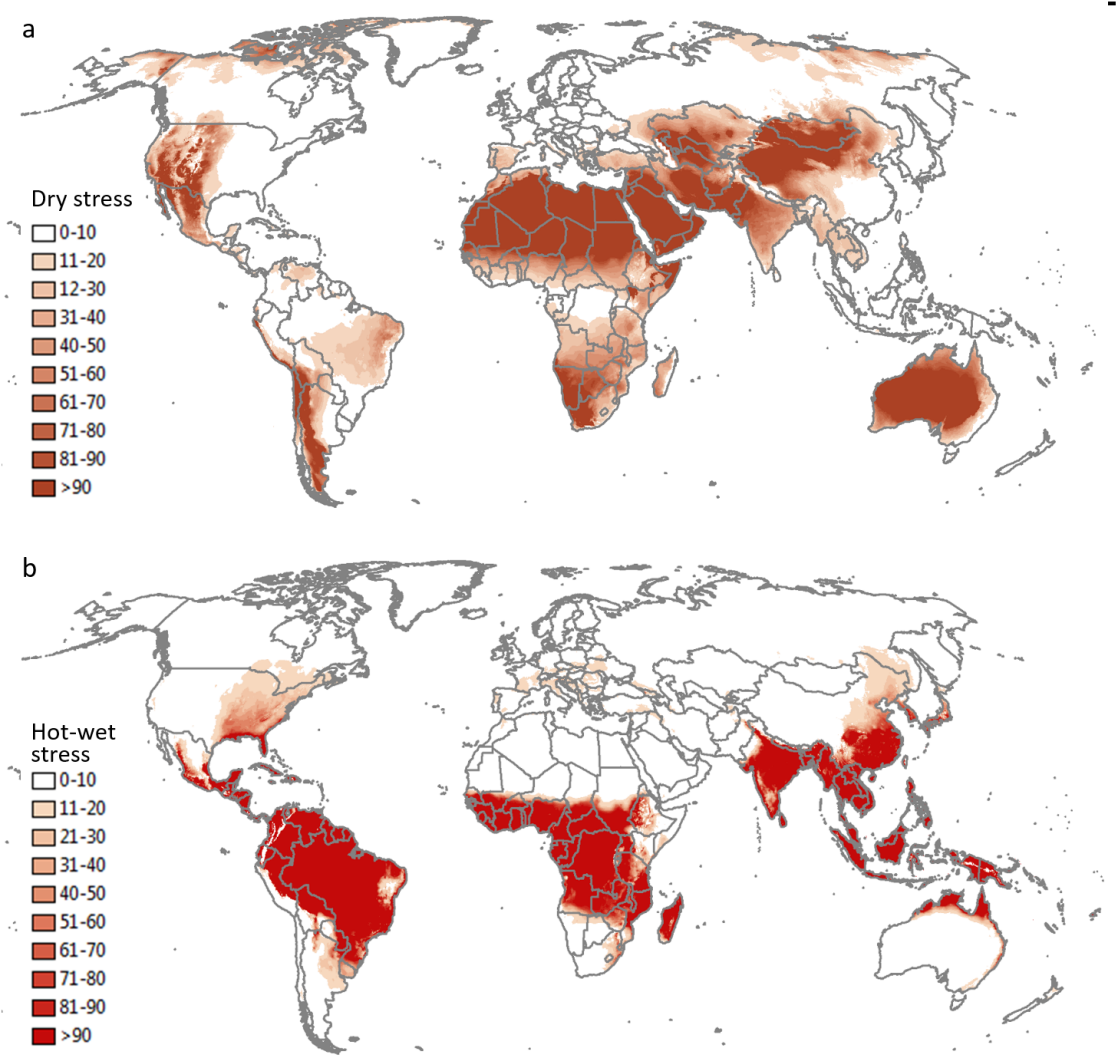
**Annual (a) dry stress (DS) and (b) hot-wet stress (HWS) indices for *V*. *germanica***.

The potential distribution of *V*. *germanica* in Argentina under a natural rainfall scenario is shown in Fig 2A. Many of the presence sites in the Patagonian region fall out of the modelled potential range. With the current model, the main limiting factor in this region is dry stress (Fig 3). When 2.5 mm day^-1^ irrigation was added as a top-up to natural rainfall during summer, all the presence sites fall into the suitable range, including the presence sites in the colder Andes Mountains (Fig 2B). Fig 2C gives a composite risk map, based on the areas across the globe considered to be under irrigation [47]. In this scenario, the potential range is similar to a natural rainfall scenario, with many of the presence sites falling out of the climatically suitable range. However, the composite risk scenario did show a slight improvement in model fit compared to the natural rainfall scenario, with some of the sites, e.g. in the northern region of Patagonia, now being suitable. In the Pampas region in north-eastern Argentina, the climate is estimated to be suitable, as with previous models, yet *V*. *germanica* is absent from this region.

**Fig 4:**
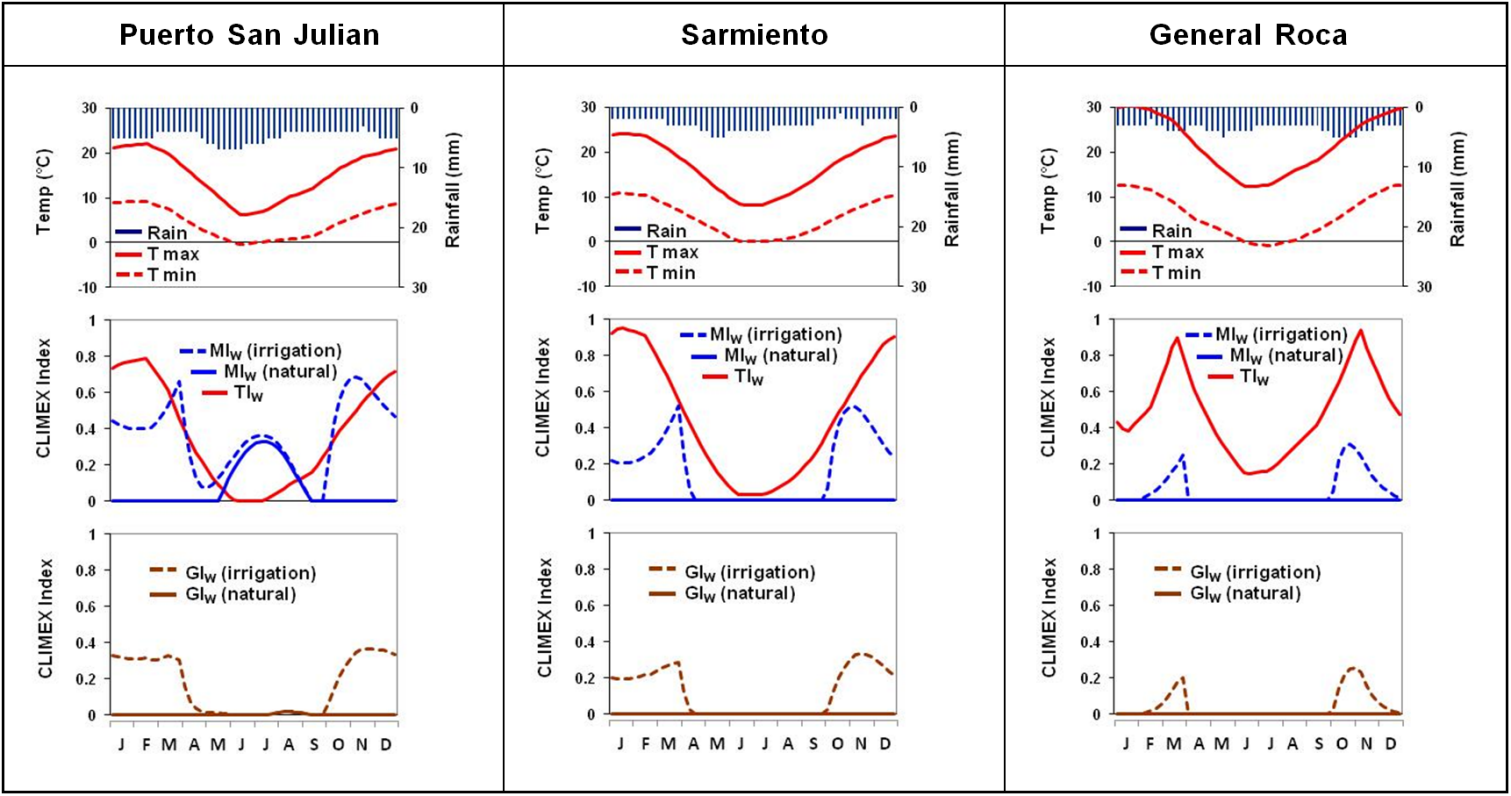
Climate and CLIMEX indices for three sites in the Patagonian region in Argentina where *V*. *germanica* occurs, but is projected to be climatically unsuitable without irrigation. T max = maximum temperature; T min = minimum temperature; MI_W_ (irrigation) and GI_W_ (irrigation) = weekly moisture and growth indices under an irrigation scenario (2.5 mm day^-1^ top-up to natural rainfall during summer) respectively; MI_W_ (natural) and GI_W_ (natural) = weekly moisture and growth indices under a natural rainfall scenario respectively; TI_W_ = weekly temperature index.

Fig 4 shows the seasonal climate and CLIMEX indices in selected locations in the Patagonian region (see Fig 1 for positions of these locations). In all three these locations, the GI_W_ was zero in the absence of irrigation. However, when irrigation was applied, the GI_W_ improved, fitting well with the observed phenology of the species, with queens initiating the colonies during spring and worker activity peaking during March, where after there is a decline towards the winter period, during which the species is absent.

**Fig 5:**
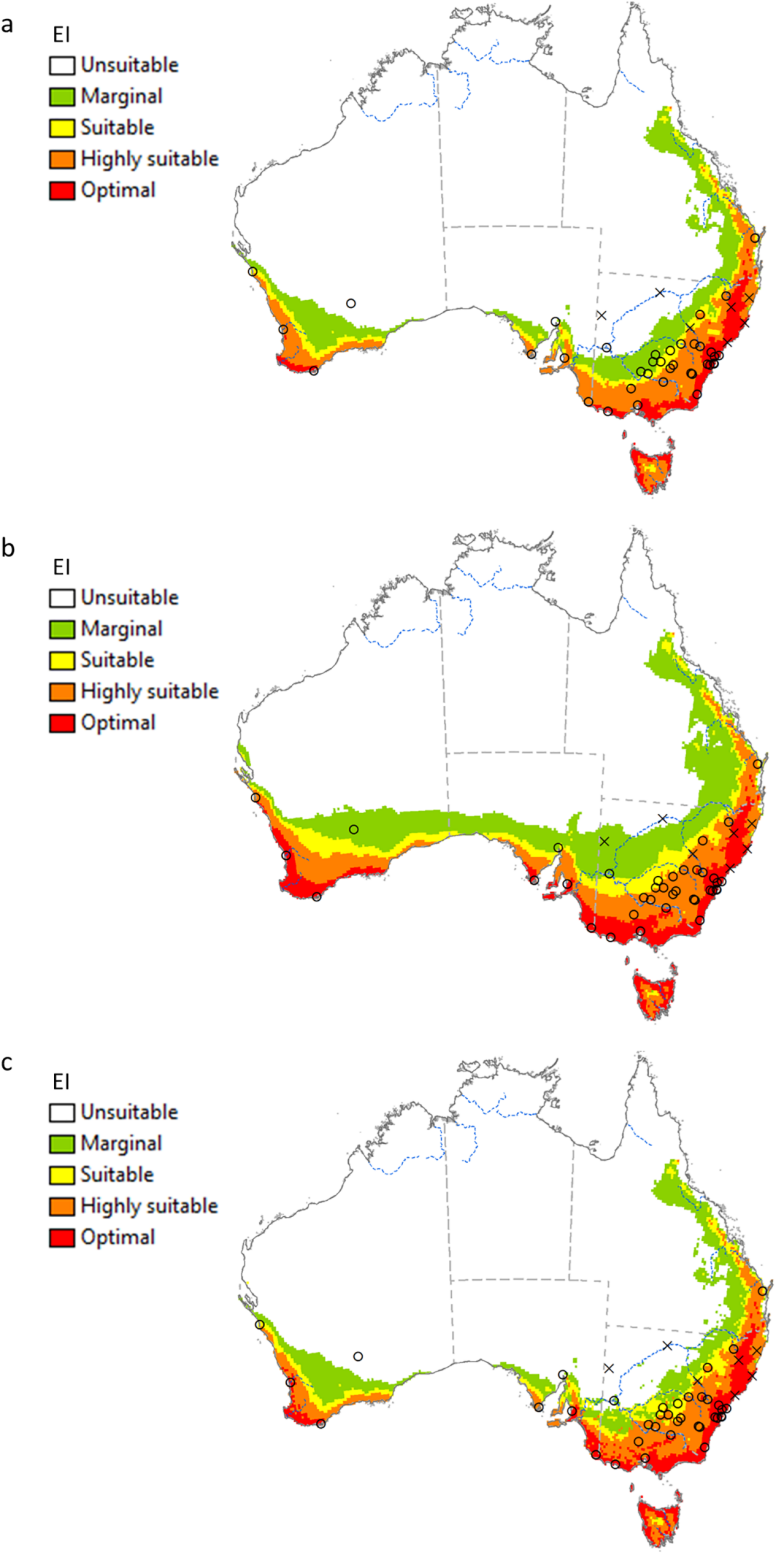
**The projected climate suitability for *V*. *germanica* in Australia, (a) without irrigation, (b) with 2.5 mm day**^**-1**^ **top-up irrigation during summer and (c) with a composite risk irrigation scenario (where areas are not under irrigation, the EI of the natural rainfall scenario is mapped, while with areas under irrigation the EI of the irrigation scenario is mapped), using the CLIMEX Ecoclimatic Index (EI).** Open circles: presence sites; black crosses: absence sites (distribution data for Tasmania is not shown); blue dotted lines: main rivers. Unsuitable: EI = 0; marginal: EI = 1–4; suitable: EI = 5–9; highly suitable: EI = 10–29; optimal: EI = 30–100.

The potential distribution of *V*. *germanica* in Australia under a natural rainfall scenario is shown in Fig 5A. The most northern sites along the west and east coast (Kalbarri and Maryborough respectively) fall into the modelled suitable range (see Fig 1 for position of the sites). However, Kalgoorlie, Port Augusta and Dareton fall out of the suitable range. This was due to dry stress (Fig 3). When the irrigation scenario was applied, these sites became climatically suitable (Fig 5B). The Darling River is also considered to be a cut-off point in the distribution of the species in New South Wales (Marc Widmer, pers comm.). This matched the modelled potential range under an irrigation scenario. However, when only the areas that are considered to be under irrigation were taken into account (composite risk scenario), Kalgoorlie, Port Augusta and Dareton fall out of the modelled suitable range (Fig 5C).

**Fig 6:**
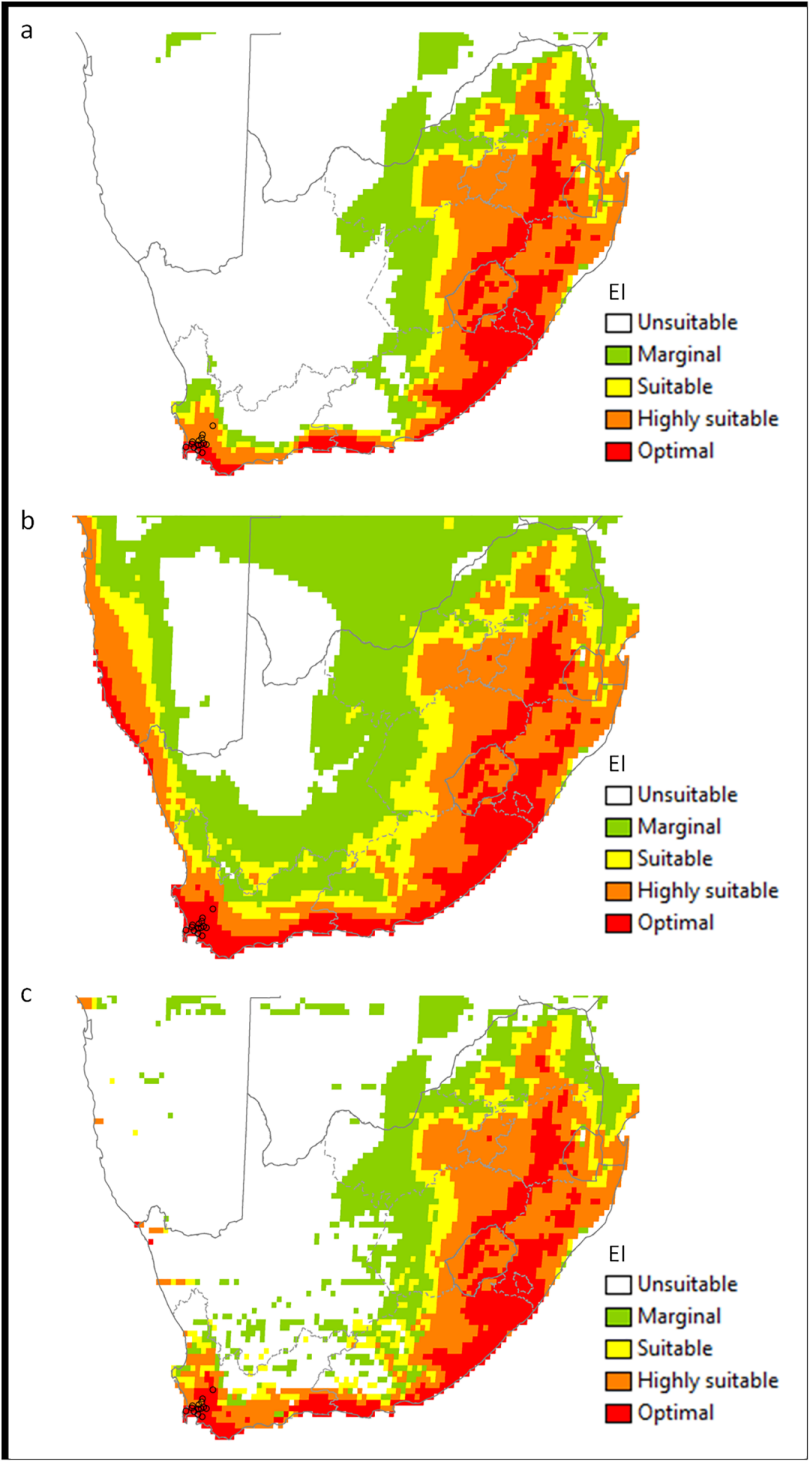
**The projected climate suitability for *V*. *germanica* in South Africa, (a) without irrigation, (b) with 2.5 mm day**^**-1**^ **top-up irrigation during summer and (c) with a composite risk irrigation scenario (where areas are not under irrigation, the EI of the natural rainfall scenario is mapped, while with areas under irrigation the EI of the irrigation scenario is mapped), using the CLIMEX Ecoclimatic Index (EI).** Open circles: presence sites. Unsuitable: EI = 0; marginal: EI = 1–4; suitable: EI = 5–9; highly suitable: EI = 10–29; optimal: EI = 30–100.

The modelled potential distribution for South Africa under a natural rainfall scenario indicates that the locations in the Western Cape where *V*. *germanica* currently occurs are where the climate is projected to be either highly suitable or optimal (Fig 6A). Suitability is also projected along a narrow band in the south coast, stretching all the way to the east coast. Most of the eastern half of the country is projected to be climatically suitable. When the irrigation scenario was applied, the climatic suitability of the region in the Western Cape in which the species occurs changed to optimal. In addition, the suitability in the Western Cape showed a northward expansion, including a large part of the Northern Cape (Fig 6B). With the composite risk scenario, the climatically suitable area is smaller than when irrigation is applied throughout South Africa, with only small patches in the Northern Cape being favourable. However, it still shows a wider potential distribution than with a natural rainfall scenario (Fig 6C).

**Fig 7:**
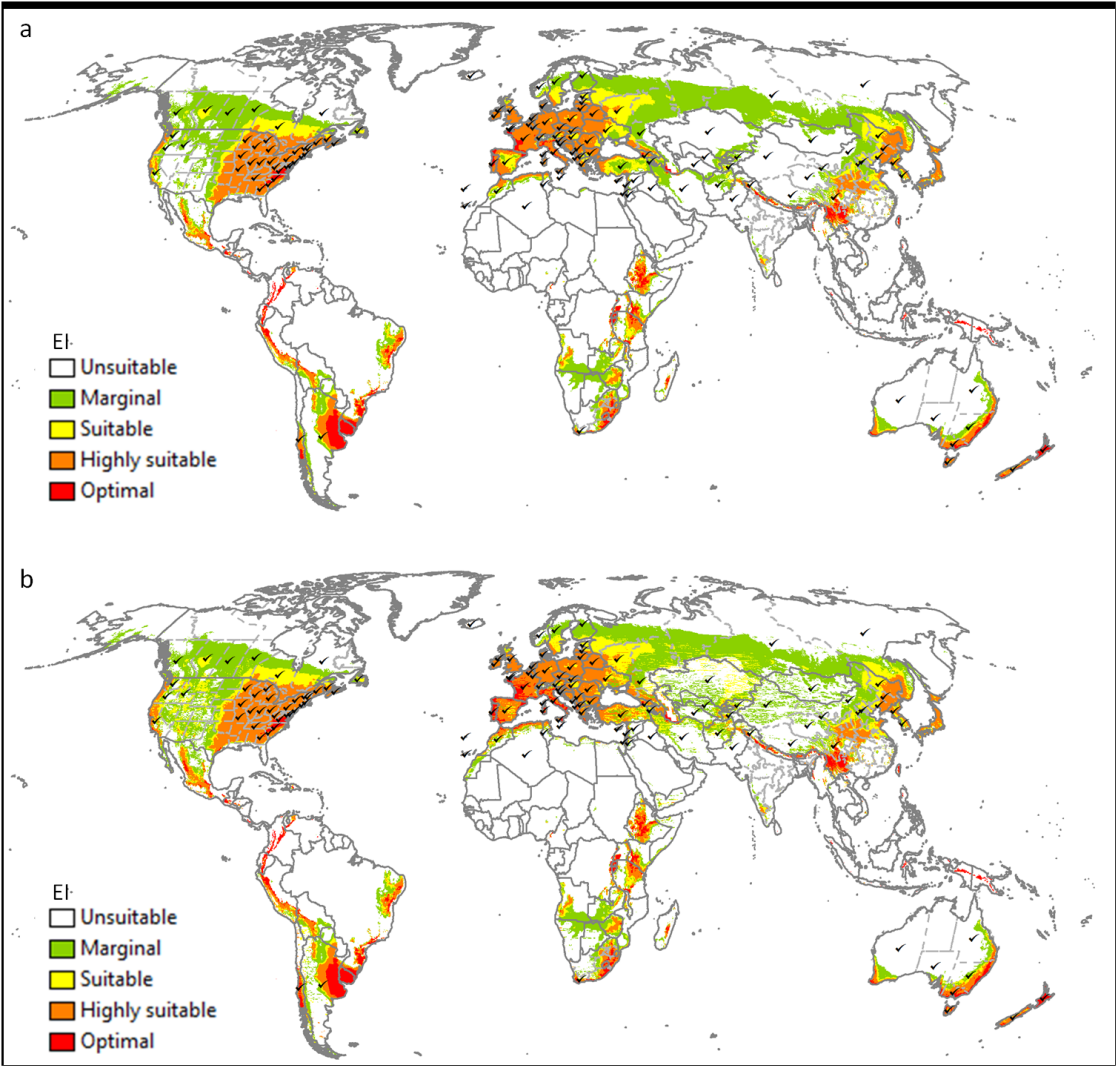
**The projected global climate suitability for *V*. *germanica*, (a) without irrigation, (b) with a composite risk irrigation scenario (where areas are not under irrigation, the EI of the natural rainfall scenario is mapped, while with areas under irrigation the EI of the irrigation scenario [2.5 mm day**^**-1**^ **top-up irrigation during summer] is mapped), using the CLIMEX Ecoclimatic Index (EI).** Tick marks: countries or provinces/states within countries where *V*. *germanica* is known to occur. Unsuitable: EI = 0; marginal: EI = 1–4; suitable: EI = 5–9; highly suitable: EI = 10–29; optimal: EI = 30–100.

Fig 7 shows the potential global distribution for a natural rainfall scenario and a composite risk scenario. Under natural rainfall, the estimated distribution of *V*. *germanica* in Asia does not provide a good fit with the actual distribution (Fig 7A). However, when an irrigation scenario is applied to those areas known to be irrigated (composite risk scenario), the fit in Asia improves dramatically (Fig 7B). Besides dry stress, hot-wet stress is also an important factor limiting the species’ distribution, with most tropical regions being climatically unsuitable (Figs 3B and 7).

## Discussion

Under a natural rainfall scenario, the model shows similarities with the models by Spradbery & Maywald [37], Tribe & Richardson [38] and Sutherst et al. [39]. In Argentina, the main difference lies in the narrow band along the Andes Mountains that now appears to be climatically suitable. This is due to the lower temperature threshold that was chosen in the current model. The fact that presence and absence data was available for Argentina made it possible to improve the current model compared with the previous three models. In Australia, there was also an improvement between the presence data and the estimated range, with the current model being similar to the models by Spradbery & Maywald [37] and Sutherst et al. [39], with less sites falling out of the estimated range than with the model by Tribe & Richardson [38]. In both these countries, the fit between presence data and the estimated range was improved when irrigation was added. However, when a composite risk scenario was mapped, where only the areas considered to be under irrigation [47] was shown, the fit was less accurate, especially in Argentina, with many presence sites in the Patagonian region falling out of the estimated suitable range.

The anomalies between distribution in Argentina and Australia and the modelled range with the composite risk scenario could be due to the dataset of Siebert et al. [47] failing to capture all areas under irrigation. However, since these presence sites in Australia and Argentina mostly fall into areas where crop production is not practiced on a commercial scale and which are unlikely to be irrigated, it is more likely that the Siebert et al. [47] dataset does not capture the effect of human habitat modification (e.g. people watering gardens, thereby supplying a water source) and the geography of the areas (e.g. the fact that many presence sites in Argentina lie along river beds where there will be an increase in water availability) on the distribution of the species. These few anomalies may therefore lie outside the limits of the methods to estimate pest risks and habitat suitability. The effects of agricultural irrigation may also be confounded with other anthropogenic activities, which could provide food and shelter for *V*. *germanica*. However, these factors operate at a scale finer than the agro-climatic scale. The irrigation practices directly affect the potential for a broad range of plant hosts to persist in xeric environments, and the polyphagous nature of *V*. *germanica* means that it is unlikely to be limited within these irrigated regions that are modelled as being climatically suitable. Conversely, there may be some isolated locations outside of the agricultural irrigation zones that support populations of *V*. *germanica* due solely to the presence of anthropogenic activities.

The Pampas region, which is projected to be climatically suitable, is a fertile agricultural region. Besides suitable climate, there should also be a sufficient food source available. Therefore, it is unclear why the species is unknown from this region. However, there are some small mountain ranges (e.g. Sierra Lihuel-Calel, Sierra de la Ventana and Sierra del Tandil) separating the Patagonian region from the Pampas region, which may have acted as a geographical barrier, so far preventing or slowing *V*. *germanica* from spreading into the Pampas region.

In Argentina, the improved fit between GI_W_ and the seasonal phenology of *V*. *germanica* with irrigation indicated a need for sufficient water supply. The latter was observed by Horwood et al. [40], who found a positive correlation between rainfall and wasp abundance in the Sydney Metropolitan Area, Australia. Horwood et al. [40] suggested that rainfall is needed as a source for drinking water, as well as for the formation of wood pulp used in nest construction. In Tasmania, Madden [48] also indicated a positive effect of autumn and spring rainfall on queen production and nest establishment respectively. It was suggested that autumn rain leads to an increase in the activity of insects, while spring rain increases both insect activity and flowering, leading to a larger supply of proteins and carbohydrates [48]. This supports the inclusion of an irrigation scenario, which simulates the effect of rainfall in an otherwise dry region with insufficient water supply.

Some authors stated that excess rain can negatively impact the species by flooding of the underground nests, preventing either survival of overwintering nests in the warmer climates (autumn rain) or survival of the newly founded nests (spring rain) [37, 48, 49]. However, in Australia, New Zealand and the USA, they were found to also nest aboveground [5, 11, 25, 37], with some areas in Southern Australia having a larger proportion of nests aboveground than underground [31, 46]. Kasper et al. [31] attributed this to the possibility that more nesting sites are available aboveground, e.g. in highly populated areas where lawns and meadows are replaced by buildings, roads and concrete. In North America, more nests were constructed aboveground compared to Europe [5]. The tendency to nest aboveground is also observed in South Africa. In such instances, the wasps will not drown when the soil becomes saturated.

In South Africa, the estimated suitable range under a natural rainfall scenario showed similarities to previous models [37–39]. If climate is the only factor taken into consideration, *V*. *germanica* will be able to spread easily along the coastal band in the south into the favourable zone in the eastern part of the country. Tribe & Richardson [38] also considered this to be the most likely route for natural expansion of the species’ range. When irrigation is considered with the composite risk scenario, the climatic suitability increased along the southern coastal band, shifting more towards the north, making this an even more favourable route of expansion. Furthermore, some additional locations in the northern parts of the Western Cape, as well as the southern parts of the Northern Cape were estimated to be suitable, although mostly these were marginally suitable. This may be a pathway for jump dispersal due to human transport, making it easier for the species to expand its range into the more favourable eastern zone. It also gives an indication that a trapping network for detection of the species outside its current range in the Western Cape should not only include the favourable coastal band and eastern zone, but also extend northwards into the Northern Cape. It is clear that the realised distribution range of *V*. *germanica* in South Africa is still small relative to its potential range. Based on this information we suggest a co-ordinated eradication effort can at least be considered.

At a global scale our model with a natural rainfall scenario showed similarities to the models by Spradbery & Maywald [37] and Sutherst et al. [39]. However, the model by Tribe & Richardson [38] estimated the species to be better adapted to the humid tropical regions in Africa and south-eastern Asia, including the Philippines, Indonesia, Malaysia and Papua New Guinea and south-eastern China, where the species is absent. The main reason for this difference with the other two models, as well as the current model, is because the model described in Tribe & Richardson [38] did not include hot-wet stress (Fig 3B), which was needed to model South East China as unsuitable. With irrigation (composite risk scenario), the improved fit between the current distribution and the estimated range again supported the inclusion of irrigation when modelling the potential distribution of the species. The main areas still at risk of invasion by *V*. *germanica* include the western region of the USA, Mexico, small areas in Central America and in the north-western region of South America, eastern Brazil, western Russia, north-western China, Japan, the Mediterranean coastal regions of North Africa, and parts of southern and eastern Africa.

By including agricultural irrigation as a location-specific factor we were able to markedly improve the overall fit of the model, simultaneously improving both sensitivity and specificity. This modelling technique [50–52] is likely to yield significant improvements to niche models of most agricultural pests, diseases and weeds.

## Supporting information

S1 File Underlying data. (XLSX)
